# Employment Probability Trajectories Up To 10 Years After Moderate-To-Severe Traumatic Brain Injury

**DOI:** 10.3389/fneur.2018.01051

**Published:** 2018-12-05

**Authors:** Emilie I. Howe, Nada Andelic, Paul B. Perrin, Cecilie Røe, Solrun Sigurdardottir, Juan Carlos Arango-Lasprilla, Juan Lu, Marianne Løvstad, Marit Vindal Forslund

**Affiliations:** ^1^Department of Physical Medicine and Rehabilitation, Oslo University Hospital, Oslo, Norway; ^2^Institute of Clinical Medicine, Faculty of Medicine, University of Oslo, Oslo, Norway; ^3^Research Centre for Habilitation and Rehabilitation Models and Services (CHARM), Faculty of Medicine, Institute of Health and Society, University of Oslo, Oslo, Norway; ^4^Department of Psychology, Virginia Commonwealth University, Richmond, VA, United States; ^5^Department of Research, Sunnaas Rehabilitation Hospital Trust, Nesoddtangen, Norway; ^6^BioCruces Health Research Institute, Cruces University Hospital Barakaldo, Barakaldo, Spain; ^7^IKERBASQUE, Basque Foundation for Science, Bilbao, Spain; ^8^Division of Epidemiology, Department of Family Medicine and Population Health, Virginia Commonwealth University, Richmond, VA, United States; ^9^Department of Psychology, University of Oslo, Oslo, Norway

**Keywords:** brain injury, outcome assessment, prospective studies, return to work, rehabilitation

## Abstract

**Aims:** To examine trajectories of employment probability up to 10 years following moderate-to-severe traumatic brain injury (TBI) and identify significant predictors from baseline socio-demographic and injury characteristics.

**Methods:** A longitudinal observational study followed 97 individuals with moderate-to-severe TBI for their employment status up to 10 years post injury. Participants were enrolled at the Trauma Referral Center in South-Eastern Norway between 2005 and 2007. Socio-demographic and injury characteristics were recorded at baseline. Employment outcomes were assessed at 1, 2, 5, and 10 years. Hierarchical linear modeling (HLM) was used to examine employment status over time and assess the predictors of time, gender, age, relationship status, education, employment pre-injury, occupation, cause of injury, acute Glasgow Coma Scale (GCS) score, duration of post-traumatic amnesia (PTA), CT findings, and injury severity score, as well as the interaction terms between significant predictors and time.

**Results:** The linear trajectory of employment probabilities for the full sample remained at ~50% across 1, 2, 5, and 10-years post-injury. Gender (*p* = 0.016), relationship status (*p* = 0.002), employment (*p* < 0.001) and occupational status at injury (*p* = 0.005), and GCS (*p* = 0.006) yielded statistically significant effects on employment probability trajectories. Male gender, those in a partnered relationship at the time of injury, individuals who had been employed at the time of injury, those in a white-collar profession, and participants with a higher acute GCS score had significantly higher overall employment probability trajectories across the four time points. The time^*^gender interaction term was statistically significant (*p* = 0.002), suggesting that employment probabilities remained fairly stable over time for men, but showed a downward trend for women. The time^*^employment at injury interaction term was statistically significant (*p* = 0.003), suggesting that employment probabilities were fairly level over time for those who were employed at injury, but showed an upward trend over time for those who had been unemployed at injury.

**Conclusion:** Overall employment probability trajectories remained relatively stable between 1 and 10 years. Baseline socio-demographic and injury characteristics were predictive of employment trajectories. Regular follow-up is recommended for patients at risk of long-term unemployment.

## Introduction

The majority of individuals with traumatic brain injuries (TBIs) in high-income countries survive due to improvements in overall trauma care (1). Most survivors are of working age (2), and one of the challenges for this group is to return to work and maintain employment over time (3–6). The participation in employment represents a key rehabilitation goal after TBI in order to avoid the personal and socio-economic burden of unemployment. Identifying early prognostic factors associated with employment and employment probability trajectories can help identify persons who are at risk of unemployment and to alleviate the burden of TBI through more effective vocational rehabilitation programs.

Despite substantial research regarding employment outcomes and their prognostic factors (7–13), there are few studies looking at employment probability from a long-term perspective after TBI (i.e., 10 years after injury) (14). Ponsford et al. (15) examined aspects of functioning affected by complicated mild to severe TBI over a span of 10 years and found that only half of the sample returned to previous leisure activities and fewer than half were employed at each follow-up post-injury (2, 5, and 10 years). More recently, Cuthbert et al. (16) studied the 10 years patterns of employment in working age persons with moderate-to-severe TBIs who were discharged from a Traumatic Brain Injury Model Systems (TBIMS) center in the United States. They used a generalized linear mixed model, and included 1, 2, 5, and 10 years follow-ups. Results indicated that age, gender, cultural factors, education, duration of post-traumatic amnesia (PTA), and pre-injury substance abuse significantly predicted the trajectory of post-injury employment. The authors concluded that the overall decline in trajectories of employment probability between 5 and 10 years post-injury may suggest the chronic effects of TBI, and the influence of national and labor market forces on employment outcome. Similarly, Grauwmeijer et al. (14) evaluated the predictors and probability of employment over a 10 years period (3, 6, 12, 18, 24, and 36 months and 10 years post-TBI) in a Dutch sample of moderate-to-severe TBIs using generalized estimating equations and a logistic regression analysis. The authors concluded that 10 years employment probability is related to time, severity of injury and pre-injury employment. After an initial increase in the first 2 years post TBI, the employment probability stabilized at 57% after 2 years and decreased to 43% in the long-term (14), in line with the study by Cuthbert et al. (16).

Taken together, in addition to the socio-demographics and injury related characteristics, differences in governmental policies, health care and welfare systems, rehabilitation services, and culture may influence the predictors of employment trajectories (5, 13, 16–19). Thus, studies from different countries are required to provide a better understanding of factors influencing the employment probability and needs of rehabilitation and long-term follow-up programs.

We previously reported the employment probability trajectories up to 5 years post-injury (5) by using multi-level modeling, and found fairly constant employment rates of ~50% across the three follow-up time points at 1, 2, and 5 years post-TBI. Being single, unemployment at the time of injury, blue collar occupation, lower GCS score at hospital admission, and longer duration of PTA were significant predictors of unemployment at 1, 2, and 5 years post-injury.

This study is an extension which aims to examine employment probability trajectories up to 10 years after moderate-to-severe TBI, and to investigate whether those trajectories could be predicted by socio-demographics and injury characteristics. Based on the previously mentioned studies from the US and Netherlands, we hypothesized that the employment probability would decrease from 5 to 10 years post-injury.

## Materials and Methods

### Participants

A longitudinal cohort study was conducted including patients with acute TBI who had been admitted from 2005 to 2007 to the Trauma Referral Centre for the South-Eastern region of Norway, covering a population of nearly 2.6 million people. Patients were assessed in the acute phase (baseline) and followed up at 1, 2, 5, and 10 years after injury. Inclusion criteria were (a) age 16–55 years, (b) residence in eastern Norway, (c) admission with ICD-10 diagnosis S06.0–S06.9 within 24 h of injury, and (d) presence of moderate-to-severe TBI with a Glasgow Coma Scale (GCS) (20) score of 3–12 at admission or before intubation. Exclusion criteria were (a) previous neurological disorders/injuries, (b) associated spinal cord injuries, (c) previously diagnosed severe psychiatric or substance abuse disorders, and (d) unknown address or incarceration. For additional details, see study by Forslund et al. (5).

**Figure 1:**
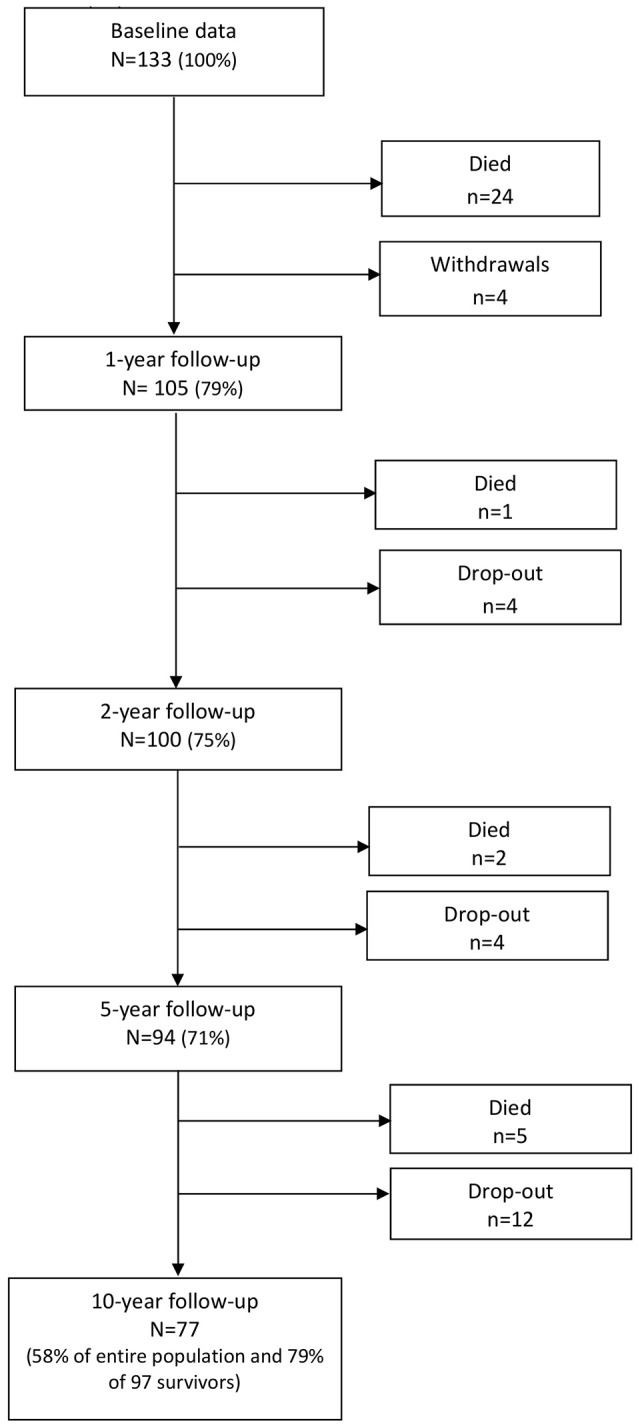
Flowchart.

Overall, 133 individuals met the inclusion criteria. Thirty-two patients died during the acute or post-acute phase and four withdrew, leaving 97 survivors analyzed in this study (see Figure 1). The overall attrition rate in the surviving population was 21%. Because full information maximum likelihood (FIML) estimation was used to account for missing data at the various follow-ups, all participants were able to be retained in the model, generating statistical estimates that were unbiased due to attrition.

### Measures

The outcome variable in this study was employment status at 1, 2, 5, and 10 years after injury. Employment was dichotomized into employed and unemployed, where individuals in the employed group consisted of individuals working full/part time or studying (high school, college, or university), while members of the unemployed group were jobseekers, on sick leave or work assessment allowance, or receiving disability pension. Working or studying full time was equal to 37.5 productive hours per week (i.e., 100% in Norway), while part-time employment was defined as working <37.5 h per week.

The independent variables (predictors) used in this study were: Gender (male vs. female), age at time of injury (in years), relationship status at hospital admission (partnered [married/cohabitant] vs. single), education (≤12 years vs. >12 years), employment status at time of injury (employed vs. unemployed), occupation prior to admission [blue collar (physical work) vs. white collar (non-physical work/being a student)], acute GCS (continuous), cause of injury (traffic accident vs. other), length of PTA (number of days) measured by the Galveston Orientation and Amnesia Test (GOAT) (21), Injury Severity Score [ISS; range from 1 to 75 (best to worst)] (22), and CT severity score. All patients had an acute CT head scan followed by a second control scan between 6 and 12 h after the injury. All CT scans were assessed and categorized by the same neuroradiologist according to the Marshall CT classification (23). The CT scan that showed the most extensive degree of intracranial damage (i.e., the largest hematoma thickness/midline shift and/or with the most extensive degree of parenchymal damage) within the first 24 h was used for classification.

### Procedure

Pre-injury and injury-related characteristics from the acute phase were extracted from medical records. At the 1, 2, 5, and 10 years follow-ups, a physiatrist performed the assessments and interviews of patients at the outpatient department. Several patients made requests that the assessments and interviews should be conducted by telephone, and this was complied with. The study was approved by the Regional Committee for Medical Research Ethics, East Norway, and the Norwegian Data Inspectorate. All participants gave their written informed consent to participate in the study.

### Data Analysis

Descriptive statistics were used to present demographics and injury related variables, and results are presented as percentages and means with standard deviations (SD) as appropriate. Hierarchical linear modeling (HLM) was used to examine trajectories of employment probability across 1, 2, 5, and 10 years after injury and identify baseline predictors. HLM was selected so that a full trajectory across all four time points could be analyzed and predicted, as opposed to separate and limited predictions of employment probability at each independent time point. A conditional (null) model was run first to determine whether there was sufficiently large clustering of employment probability variance within participants to proceed with HLM. Unconditional growth linear (straight line), quadratic (U-shaped), and cubic models (S-shaped) were then run with no predictors to determine the most accurate model for linear or polynomial (curved) architecture of employment probabilities over time.

Once the most accurate curvature model was identified, predictors were entered simultaneously as fixed effects into an HLM after being centered or given a reference point of 0, along with time (given that linear trajectories of employment probabilities were found, outlined below). The HLM determined whether linear trajectories of employment probabilities across the four time points could be predicted by the demographic and injury characteristics of time [coded as 0 (1 year), 1 (2 years), 4 (5 years), or 9 (10 years) to reflect actual spacing between time points], gender (1 = female, 0 = male), age, relationship status (1 = partnered, 0 = single), education (1 = >12 years, 0 = ≤12 years), employment at admission (1 = employed, 0 = unemployed), occupational status (1 = white collar, 0 = blue collar), continuous GCS score, cause of injury (1 = motor vehicle, 0 = not motor vehicle), length of PTA (days), CT severity score, and ISS. A second HLM included the significant predictors identified from the full HLM, the variable of time, and interaction terms between the variable of time and the significant predictors.

## Results

The mean age of the 97 patients at the time of injury was 30.3 (SD = 10.8) years, 76% were men and 60% were injured in traffic accidents. The mean GCS at hospital admission was 7.2 (SD = 3.2). Of all patients, 73% received inpatient rehabilitation with mean length of stay 59 days (SD = 37 days). Demographics and injury-related characteristics are presented in Table 1.

**Table 1:** Demographics at time of injury and injury characteristics.

**Variable**	***n* (%)**	**Mean (SD)**	**Total *n***
Age at injury in years		30.3 (10.8)	97
Gender			97
Male	76 (78.4)		
Female	21 (21.6)		
Relationship status			97
Partnered	28 (28.9)		
Single	69 (71.1)		
Education level			96^*^
≤ 12 years	54 (56.3)		
>12 years	42 (43.7)		
Employment status			97
Yes	80 (82.5)		
No	17 (17.5)		
Occupational status			97
Blue collar	46 (47.4)		
White collar	51 (52.6)		
Disability pension	4 (4.0)		
Injury cause			97
Traffic accident	58 (59.8)		
Other	39 (40.2)		
Glasgow Coma Scale (GCS)		7.2 (3.2)	97
Moderate (9–12)	32 (33.0)		
Severe (3–8)	65 (67.0)		
Post-traumatic amnesia (PTA) in days		26.0 (30.0)	91^**^
CT Head Marshall Score		2.6 (1.1)	97
Score 1–2	46 (47.4)		
Score 3+	51 (52.6)		
Injury Severity Score		30.0 (13.6)	97
Total acute length of stay in days		29.0 (25.0)	97
In-patient rehabilitation length of stay in days		59.0 (37.0)	71^***^

* *Missing data on 1 individual*.

** *Missing data on 6 individuals*.

*** *Only 71 individuals received in-patient rehabilitation (length of stay and mean stay is only calculated for those actually receiving it rather than the whole population)*.

Of all patients, 18% were unemployed at the time of injury (jobseekers 7%; work assessment allowance 5%; sick leave 2%; disability pension 4%). Of these, 80% were men, 60% >30 years, 70% with <12 years of education and 60% living alone.

The employment rate dropped from 82% pre-injury to 53% at 1 year follow-up and thereafter remained fairly stable up to 10 years (48, 55, and 50% at 2, 5, and 10 years follow-ups). At 10 years follow-up, 28% of the patients were in full-time jobs. Among the 22% of patients who were in part-time jobs, the majority (76%) received graded disability pension. Of the unemployed patients, 80% received full disability pension, 13% received work assessment allowance, and the remaining patients were jobseekers. A majority (79%) of the patients who were unemployed at 10 years were in the severe TBI group as measured by the GCS at injury time.

### Unconditional Model and Unconditional Growth Models

The unconditional model yielded a statistically significant estimated participant variance of 0.17 (Wald Z = 6.05, *p* < 0.001), as well as a statistically significant estimated residual variance of 0.08 (Wald Z = 11.33, *p* < 0.001). The intraclass correlation coefficient was calculated to be 0.68, indicating that ~68% of the total variance of employment probabilities was associated with the participant grouping (i.e., based on employment probability being correlated within each participant) and that the assumption of independence was violated. This suggests there was sufficiently large clustering of employment probability variance within participants to proceed with HLM. In other words, an intraclass correlation coefficient this high suggests a fairly high level to which employment probability is consistent across the same individual. The unconditional growth model was then run separately with the successive additions of time (-2LL = 321.50) quadratic time (-2LL = 321.35) and cubic time (-2LL = 315.48) in order to determine the shape of the best fitting architecture of employment probabilities over time, suggesting that a linear (straight line) trajectory best fit employment probability trajectories (The critical *X*^2^ value for significant difference at α = 0.05 is a >3.841 drop from the previous model).

### Full Model

An HLM examined whether employment probability trajectories over time could be predicted by socio-demographic and injury characteristics at the time of injury. All statistically significant and non-significant fixed effects from the full HLM and their b-weights, *p*-values, and 95% confidence intervals appear in Table 2. The linear trajectory of employment probabilities remained level over time across the full sample (e.g., no significant increase or decrease). Gender, relationship status at injury, employment at injury, occupational status, and GCS all yielded statistically significant effects on participants' employment probability trajectories.

**Table 2:** Demographic and injury predictors of employment probability trajectories across 1, 2, 5, and 10 years.

**Predictor**	***b*-weight**	***SE***	***p*-value**	**95% Confidence Interval**	
				**Lower Bound**	**Upper Bound**
Intercept	0.018	0.109	0.870	−0.198	0.234
Time	−0.002	0.005	0.642	−0.012	0.008
Gender (1 = female, 0 = male)	−0.222^*^	0.090	0.016	−0.400	−0.043
Age	−0.006	0.004	0.159	−0.015	0.002
Relationship Status (1 = partnered, 0 = single)	0.305^**^	0.097	0.002	0.112	0.498
Education	−0.045	0.050	0.367	−0.143	0.054
Employment (1 = employed, 0 = unemployed)	0.447^***^	0.097	< 0.001	0.254	0.640
Occupational Status (1 = white collar, 0 = blue collar)	0.243^**^	0.085	0.005	0.074	0.411
GCS	0.038^**^	0.014	0.006	0.011	0.065
Cause of Injury (1 = motor vehicle, 0 = not motor vehicle)	0.007	0.085	0.936	−0.161	0.175
PTA	−0.003	0.001	0.068	−0.006	0.000
CT Severity Score	−0.031	0.037	0.404	−0.104	0.042
ISS	−0.003	0.003	0.267	−0.009	0.003

* = p < 0.05;

** = p < 0.01;

*** *= p < 0.001*.

**Figure 2:**
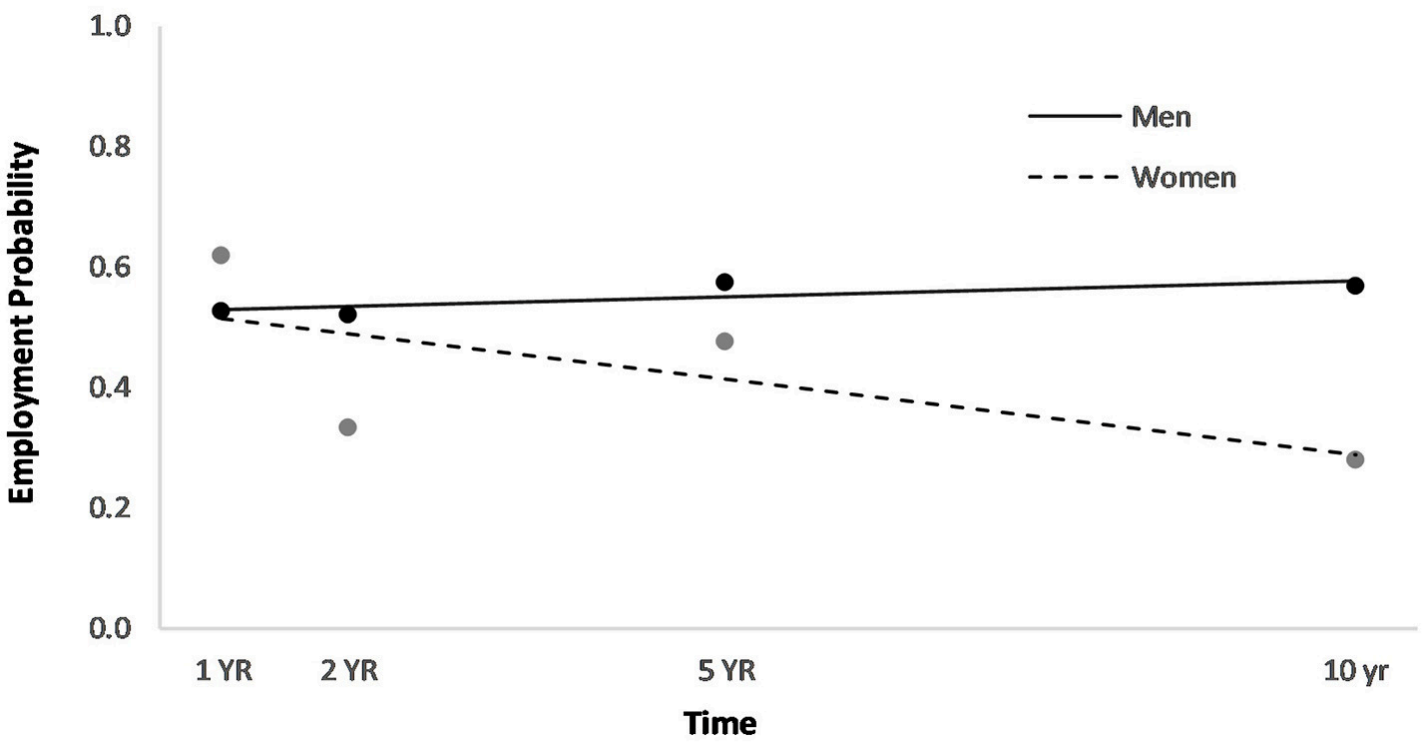
Main effect of gender on employment probability trajectories.

**Figure 3:**
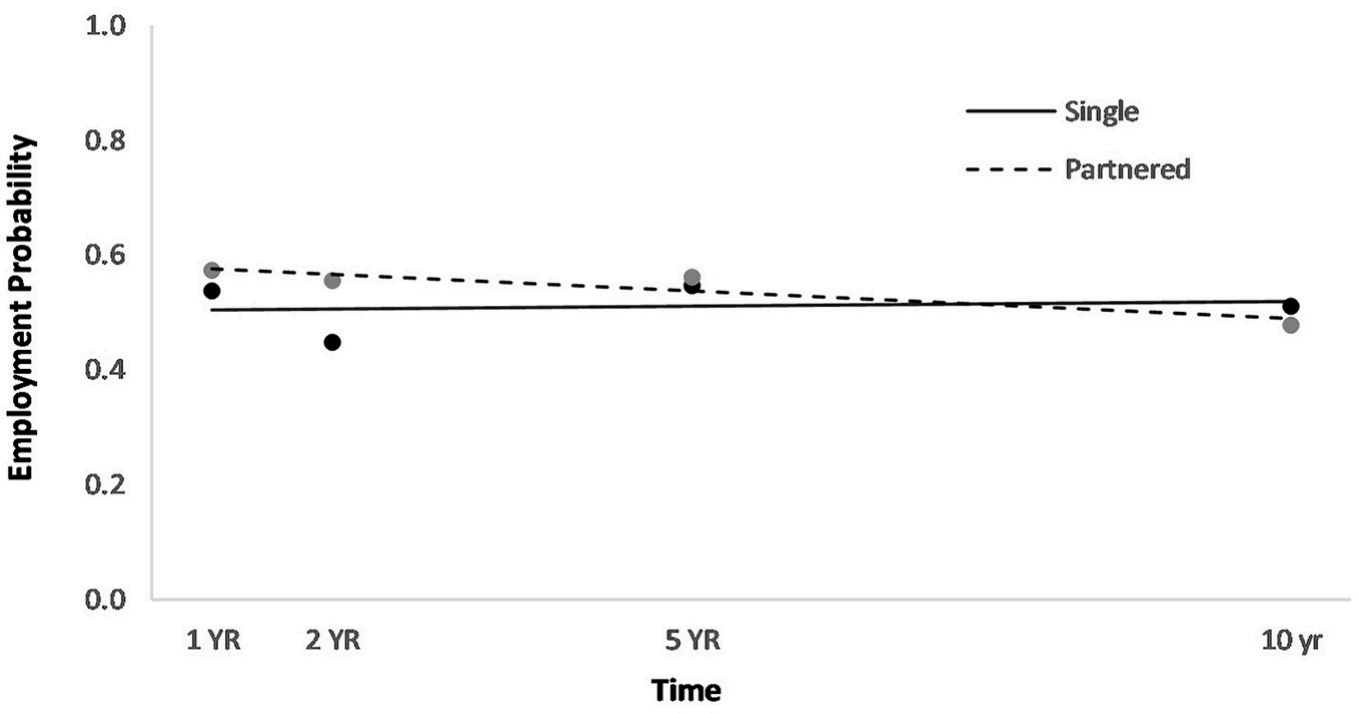
Main effect of relationship status at injury on employment probability trajectories.

**Figure 4:**
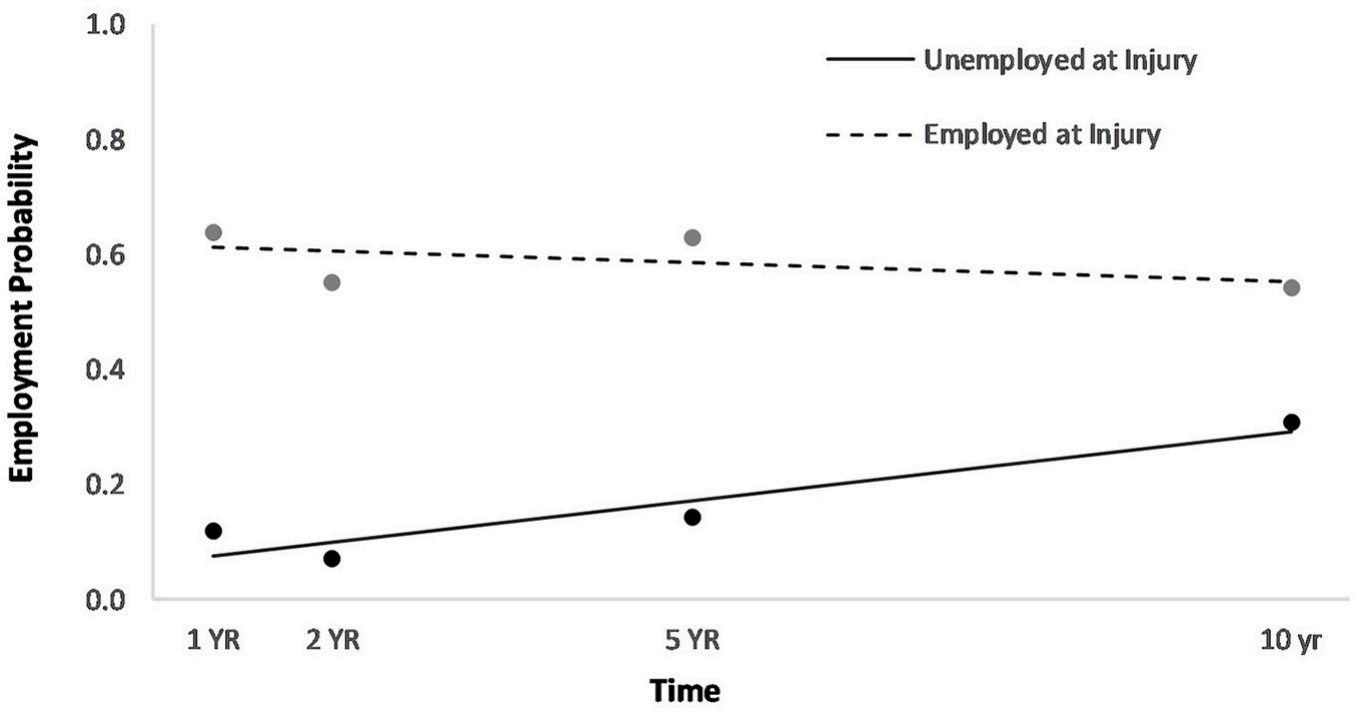
Main effect of employment at injury on employment probability trajectories.

**Figure 5:**
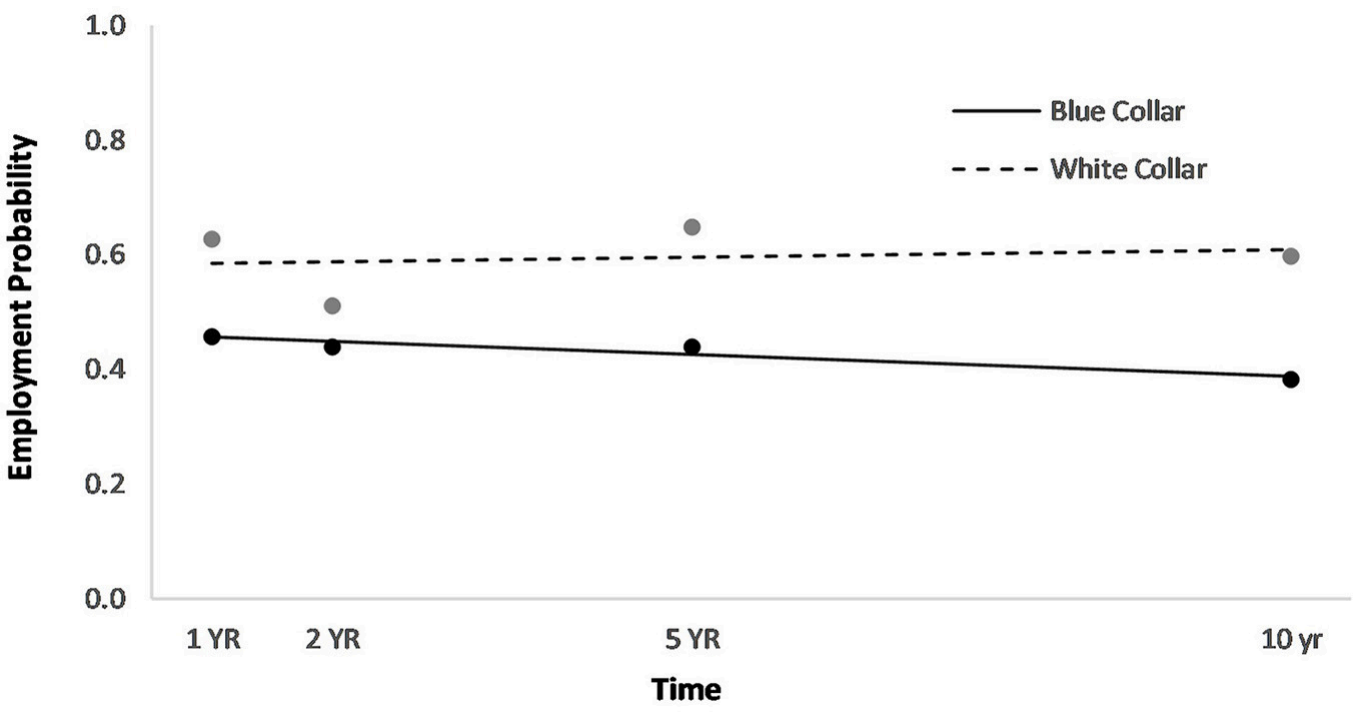
Main effect of occupational status on employment probability trajectories.

**Figure 6:**
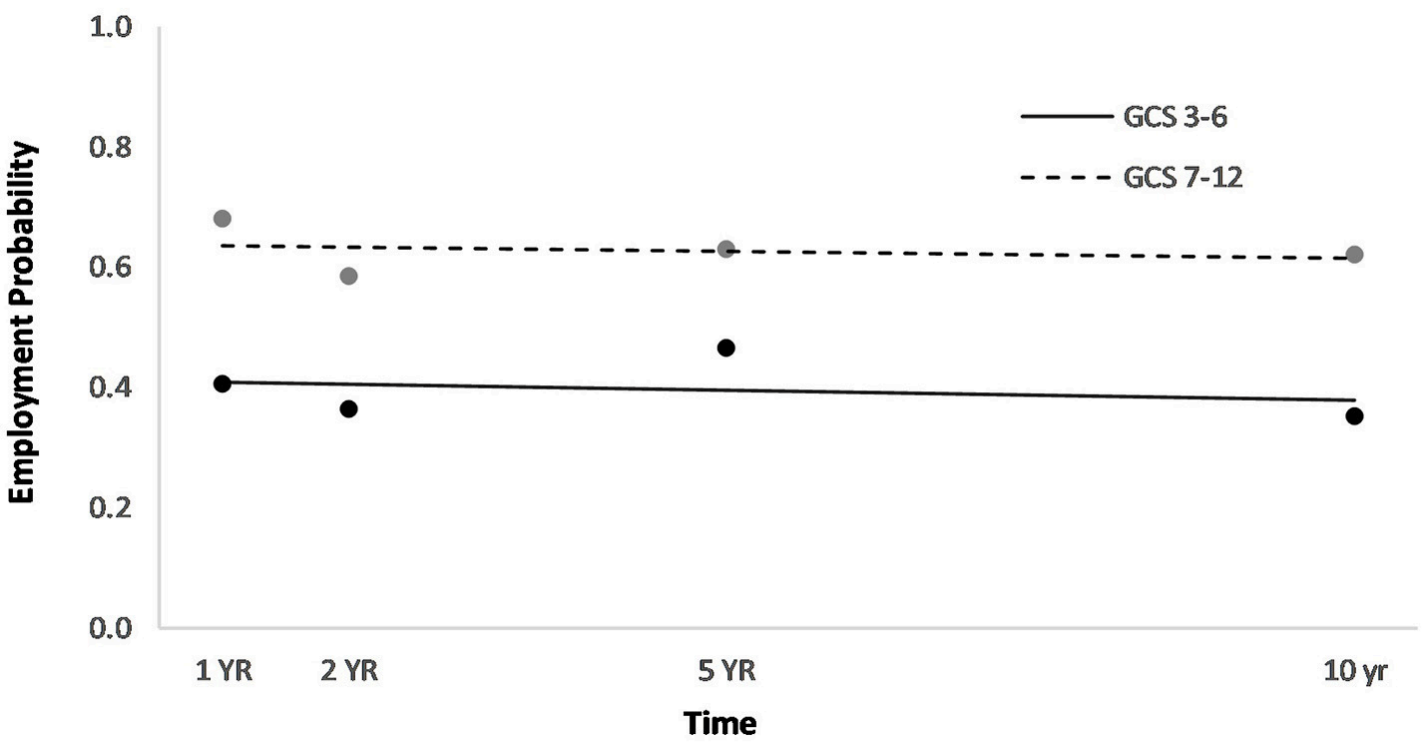
Main effect of GCS (dichotomized at mean value) on employment probability trajectories.

Men had a higher overall employment probability trajectory across the four time points compared to women (Figure 2). Individuals who had been in a partner relationship at the time of injury had a slightly higher probability trajectory of employment than those who had been single, although this effect seemed to be driven by the first three time points (Figure 3). Individuals who had been employed at the time of injury had a higher probability trajectory of employment than those who had been unemployed at injury (Figure 4). Individuals in a white collar occupation had a higher probability trajectory of employment than those in a blue collar occupation (Figure 5). Finally, participants with a lower GCS score had a lower employment probability trajectory than those with a higher score (Figure 6).

### Model With Time Interactions

An HLM examined whether employment probability trajectories could be predicted by the previously significant predictors (gender, relationship status at injury, employment at injury, occupational status, and continuous GCS), time, as well as their interactions with time (see Table 3). The time^*^gender interaction term was statistically significant (*p* = 0.002), suggesting that employment probabilities remained fairly stable over time for men but showed a downward trend over time for women (Figure 2). The time^*^employment at injury interaction term was statistically significant (*p* = 0.003), suggesting that employment probabilities were fairly level over time for those who had been employed at injury but showed an upward trend over time for those who had been unemployed at injury (Figure 4). The time^*^occupational status interaction term approached significance (*p* = 0.069) (Figure 5).

**Table 3:** Previously significant predictors and their time interaction effects on employment probability trajectories across 1, 2, 5, and 10 years.

**Predictor**	***b*-weight**	***SE***	***p*-value**	**95% Confidence Interval**	
				**Lower Bound**	**Upper Bound**
Intercept	−0.007	0.102	0.947	−0.210	0.196
Time	0.026^*^	0.012	0.033	0.002	0.051
Gender (1 = female, 0 = male)	−0.069	0.098	0.478	−0.263	0.124
Relationship Status (1 = partnered, 0 = single)	0.090	0.093	0.334	−0.094	0.274
Employment (1 = employed, 0 = unemployed)	0.532^***^	0.106	< 0.001	0.322	0.742
Occupational Status (1 = white collar, 0 = blue collar)	0.133	0.086	0.124	−0.037	0.304
GCS	0.061^***^	0.012	< 0.001	0.036	0.085
Time^*^Gender	−0.034^**^	0.011	0.002	−0.056	−0.013
Time^*^Relationship Status	−0.003	0.010	0.754	−0.024	0.017
Time ^*^Employment	−0.036^**^	0.012	0.003	−0.060	−0.012
Time^*^Occupational Status	0.018	0.010	0.069	−0.001	0.038
Time^*^GCS	−0.002	0.001	0.120	−0.005	0.001

* = p < 0.05;

** = p < 0.01;

*** *= p < 0.001*.

## Discussion

The present study is an extension of a study performed by Forslund et al. (5) which reported employment probability trajectories up to 5 years post-injury. This paper describes the 10-years trajectories and predictors of employment for 97 individuals with moderate and severe TBI.

Based on previous studies (14, 16), we hypothesized that the employment probability would decrease from 5 to 10 years post injury. Contrary to our hypothesis, the overall employment rates for the full sample remained relatively stable between 1 and 10 years at ~50% (5). The baseline employment rates were comparable to employment rates in the general population aged 25–54 years (Statistics Norway). In the past 8 years, there has been a slight decline in the employment rates in Norway. It is not possible to deduct whether the return to work process in the study population were affected by the slight general decrease in employment rates. However, even though the number of patients receiving disability pension in our study increased across the follow-ups, the percentage of jobseekers remained unchanged when comparing the baseline assessment and 10 years follow-up data.

Dahm and Ponsford (24) investigated employment trajectories after complicated mild-to-severe TBI and found an employment rate of 58% at the 10 years follow-up. Ponsford et al. (15) reported that 40% returned to open employment in some capacity and that this percentage remained stable over the first 10 years after mild-to-severe TBI in Australia. A stable employment rate across the follow-ups is probably an expression of “plateauing” of recovery after the 1st year following the injury (14, 25), but may also indicate a lack of effective, individually customized vocational rehabilitation programs aiming to improve workability and return to employment (26) such as vocational rehabilitation with supported employment (3).

Compared to the study by Grauwmeijer et al. (14), we included younger patients (age at the time of injury 16–55 years vs. 16–67 years), which may positively influence the employment probability results. The study by Cuthbert (16) included patients in the same age range as ours; however, their patients were selected from inpatient rehabilitation centers, thus representing more severe injuries which may lead to persistent, chronic consequences, with late deterioration and more unfavorable long-term outcomes. Nonetheless, methodological differences and the influence of national welfare provisions and labor market forces make it difficult to compare the employment trajectory results across countries. We can only speculate whether the demographic and injury characteristics, changes in the labor market, and welfare system differences contribute to the stable employment rates found in this study.

The following predictors were statistically significant in the models used in this study: employment at injury, relationship status, occupational status, and GCS. This is in line with results from the 5 years follow-up (5) acknowledging the importance of these factors when predicting employment outcomes after TBI. The study results demonstrated that participants who had higher GCS scores at the time of injury, and were in white-collar occupations, had significantly higher probability of being employed at all time-points. Severity of TBI (i.e., GCS score) has consistently been linked to long-term employment outcomes (5, 27, 28). Although non-significant, there was a trend toward an association between duration of PTA and employment status at 10 years. This is in accordance with previous long-term studies (16, 24), and the 1, 2, and 5-year follow-up of the current sample (5). The association between having a blue-collar occupation (i.e., manual labor) at the time of injury and post-injury unemployment is consistent with a review by Ownsworth and McKenna (29) and a study by Walker et al. (30), showing support for the association between pre-injury occupational status and employment outcomes. Being in a partner relationship at time of injury was found to significantly improve employment probability trajectories in the present study (although the effect was driven by the first time points). The results are in line with previous studies (9, 17, 31) suggesting that marital/relationship status is a significant predictor of post-injury employment.

The finding that participants who were unemployed at the time of injury were significantly less likely to be employed at each of the four time points is consistent with previous literature (5, 10, 17). A possible explanation for this finding is that previous work experience, as well as familiarity with the workplace and specific tasks, may make the transition back to work more easily achievable for those who are employed at the time of injury. Interestingly, the time^*^employment at injury interaction term was significant, suggesting that those who had been unemployed at the time of injury had an increased likelihood of being employed at the 10 years follow-up. One of the reasons may be that the majority of patients in the unemployed group were job seekers or on work assessment allowance at the time of injury, thus having the prospect of attaining jobs over time. Different workfare programs have been introduced in Norway over the last decade to meet problems in the labor market. One of the programs is the Inclusive Working Life (IW) Agreement introduced by the Norwegian Labor and Welfare Service to create a more inclusive workplace through adaptation and improvement of the work environment, reducing the utilization of sick leave and disability benefits, and retaining senior employees longer (32). The IW Agreement covers approximately 60% of the country's employees (33). However, the IW agreement has been questioned due to implementation problems and whether challenges concerning sickness related welfare consumption need to be regarded in a wider context (32).

Regarding gender differences in employment probability over time, a downward trend in employment for women was observed, while men's probabilities remained constant. The existing literature on this topic has shown mixed results (29). A study by Corrigan et al. (34) investigated changes in employment 1 year after TBI and found that women were more likely to decrease working hours or be unemployed compared to men. Fraser et al. (28) found that women were more likely than men to maintain complex work post-injury. In line with our findings, the more recent study by Cuthbert et al. (16) demonstrated a significant relationship between being female and decreased probability of employment, the same was reported in a systematic review by Willemse-Van Son (8). Possible explanations for gender-differences in employment outcome following TBI have ranged from societal influences related to gender roles, differences in job-demands, to biological differences (35). Nevertheless, there is a trend in the general population that women report more symptoms as compared to men, that there is higher percentage of women on sick leave, and that women more often have part-time jobs (36).

### Limitations and Future Directions

The current study is an extension of an existing longitudinal TBI research project. Several limitations inherent in the original design need to be acknowledged when interpreting the results. Firstly, although the study population was unselected and representative of working-age patients with moderate-to-severe TBI from the South-Eastern region of Norway, the inclusion and exclusion criteria from the original study, particularly the patients' age range at the study admission (16–55 years) and geographic setting, may limit the generalizability of the findings to a broader patient population and other healthcare settings. Secondly, the definition of employment used in this study may be a source of bias, thus limiting generalizability. Employment was categorized into unemployed (jobseekers, on sick leave or work assessment allowance, or receiving disability pension), and employed (working full-time or part-time or studying), which may have been different from other studies. Thirdly, the overall sample size for the current study is relatively small. Future studies with a larger sample size are needed to verify the findings of this study, and to account for factors other than baseline characteristics (such as functional status) which we did not assess in this study. This includes several subjective and environmental factors that may influence the employment probability such as the ability to adapt, resilience, physical, emotional and social supports, as well as access to care and current vocational rehabilitation practice. The role of work-place related factors such as possibilities for adapted work tasks, work environment, features of work organization, and the role of management also needs to be investigated to a larger degree in future research, as most TBI studies rely exclusively of individual patient characteristics. More research is needed to clarify the association between gender and interaction effects between gender and other factors on employment following TBI. Despite these limitations, the results from this study provides important insight into trajectories and predictors of employment in the long-term perspective following TBI. This information may be useful for patients, clinicians, and employment authorities and underlines the need for regular follow-ups both short- and long-term. Given the individual and societal importance of employment and return to work after TBI, future research could examine employment in more granular terms. For instance, it would be interesting to understand how the type of work, adaptations at the work place, hours worked, and/or employment stability changes over time. This would require more frequent follow-up and collecting more detailed information regarding the survivor's job situation. Better knowledge of all these factors may encourage cross-sectoral collaboration between health care services and the labor and welfare system in order to develop new individualized work-related interventions to improve both short- and long-term employment outcomes.

## Ethics Statement

This study was carried out in accordance with the recommendations of the Norwegian law on research ethics and medical Research, Regional Committees for Medical and Health Research Ethics of Norway with written informed consent from all subjects. All subjects gave written informed consent in accordance with the Declaration of Helsinki. The protocol was approved by the Regional Committee for Medical Research Ethics, East Norway, and the Norwegian Data Inspectorate.

## Author Contributions

EH, NA, CR, SS, and MF contributed to study design, data acquisition, analysis, interpretation, drafting, and finalizing the manuscript. PP contributed to analysis, interpretation, drafting, and finalizing the manuscript. JA-L, JL, and ML contributed to data interpretation, drafting, and finalizing the manuscript.

### Conflict of Interest Statement

The authors declare that the research was conducted in the absence of any commercial or financial relationships that could be construed as a potential conflict of interest.
